# Side differences and reproducibility of the Moxy muscle oximeter during cycling in trained men

**DOI:** 10.1007/s00421-024-05514-2

**Published:** 2024-05-29

**Authors:** Philip Skotzke, Sascha Schwindling, Tim Meyer

**Affiliations:** Institute of Sport and Preventive Medicine, University of Saarland, Campus B8.2, 66123 Saarbrücken, Germany

**Keywords:** Muscle oxygenation, Near-infrared spectroscopy, Physiology, Wearable

## Abstract

**Purpose:**

Portable near-infrared spectroscopy devices allow measurements of muscle oxygen saturation (SmO_2_) in real time and non-invasively. To use NIRS for typical applications including intensity control and load monitoring, the day-to-day variability needs to be known to interpret changes confidently. This study investigates the absolute and relative test–retest reliability of the Moxy Monitor and investigates side differences of SmO_2_ at the vastus lateralis muscle of both legs in cyclists.

**Methods:**

Twelve trained cyclists and triathletes completed 3 incremental step tests with 5 min step duration starting at 1.0 W/kg with an increase of 0.5 W/kg separated by 2–7 days. SmO_2_ was averaged over the last minute of each stage. For all power outputs, the intra-class coefficient (ICC), the standard error of measurement (SEM) and the minimal detectable change (MDC) were calculated. Dominant and non-dominant leg SmO_2_ were compared using a three-factor ANOVA and limits of agreement (LoA).

**Results:**

ANOVA showed no significant systematic differences between trials and side. For both legs and all intensities, the ICC ranged from 0.79 to 0.92, the SEM from 5 to 9% SmO_2_ and the MDC from 14 to 18% SmO_2_. The bias and LoA between both legs were −2.0% ± 19.9% SmO_2_.

**Conclusion:**

Relative reliability of SmO_2_ was numerically good to excellent according to current standards. However, it depends on the specific analytical goal whether the test–retest reliability is deemed sufficient. Wide LoA indicate side differences in muscle oxygenation during exercise unexplained by leg dominance.

## Introduction

One of the most important trends in endurance training in the past 10 years has been an increase in both training volume and specific training intensity made possible by a more informed and more precise load-recovery management (Sandbakk et al. 2023). With the rapidly growing field of technology in sports (Sports Tech Research Network 2023), it is predicted that the use of advanced technologies to improve objective training monitoring will continue to be one of the main trends (Sandbakk et al. 2023). Near-infrared spectroscopy (NIRS) measuring muscle oxygenation can be considered one of these technologies (Perrey 2022). Different to traditional physiological markers like heart rate, lactate or oxygen uptake which assess internal load on a systemic level, NIRS parameters give insight into the balance of oxygen delivery and oxygen demand of specific muscles non-invasively and in real-time (Barstow 2019; Perrey and Ferrari 2018). NIRS utilizes changes in the light absorbing characteristics of hemoglobin and myoglobin when oxygen is bound (Barstow 2019). Thus, oxygenated (oxy[heme]), deoxygenated (deoxy[heme]) and total hemoglobin and myoglobin (total[heme]) can be measured. The relative tissue saturation or muscle oxygen saturation (SmO_2_) can be calculated from these parameters (Feldmann et al. 2019).

Apart from lab-graded NIRS devices primarily developed to measure brain oxygenation, commercially available and less expensive portable NIRS devices dedicated to measure muscle oxygen allow the use in real-world settings and everyday training (Perrey and Ferrari 2018). One affordable portable NIRS device is the Moxy Monitor (Fortiori Designs LCC, US). The validity of the Moxy and the 0% to 100% scale has been established in active and passive trails using the arterial occlusion method (Feldmann et al. 2019). Although the Moxy has been used in various studies in applied settings (e.g. Olcina et al. 2019; Paquette et al. 2021; Pratt 2018; Yogev et al. 2023b), its reliability has not yet been adequately studied. In order to meaningfully implement the Moxy in training, reproducibility of SmO_2_, bilateral side differences and between-device measurement error need to be known. This is vital for the decision if a change between two tests is “real” or due to measurement or biological error (Chrzanowski-Smith et al. 2020). The validation study from Feldmann et al. (2019) and a few others investigated the test–retest reliability during different activities such as rest, sitting, walking and endurance exercise (Contreras-Briceño et al. 2019; Crum et al. 2017; Gandia-Soriano et al. 2022; McManus et al. 2018; Scholkmann and Scherer-Vrana 2020; Yogev et al. 2023b, 2023a). Two of those studies looked at the test–retest reliability during cycling and came to different conclusions. Yogev et al. (2023a) reported good-to-excellent relative reliability and absolute agreement between trials of 5–7% SmO_2_ for different workloads between two incremental cycling tests. Crum et al. (2017) found good reliability for low to moderate intensities, but a greater between-trial variability for higher intensities during two incremental cycling tests using the coefficient of variation (CV). The different results of the two studies can be explained by the different statistical measures used to investigate absolute reliability. For the current study, homoscedasticity has been evaluated and the correct measure for reliability chosen (Atkinson and Nevill 1998). Another shortcoming is that none of these studies reported if SmO_2_ differs between the vastus lateralis (VL) muscle of both limbs. Previous research reported side differences in power output of 5% to 20% during cycling (Carpes et al. 2010) and a greater deoxy[heme] signal amplitude in the dominant leg during counterweighted single-leg cycling (Iannetta et al. 2019). Reinpõld and Rannama (2023) found low agreement between left and right VL desaturation onset kinetics with no clear relation of these asymmetries to leg dominance. It is unclear if side differences can be observed and if SmO_2_ is different between the dominant and non-dominant leg.

This study sets out the goal to investigate the reproducibility of SmO_2_ measured by a portable near-infrared spectroscopy device at different power outputs between three—instead of the previously investigated two—cycling incremental step tests performed under similar conditions. It aims to provide information to answer two research questions: (1) What is the absolute test–retest reliability of SmO_2_ and what difference in SmO_2_ between two measurements can be considered a real change? (2) Can differences in SmO_2_ between the VL of the dominant and non-dominant leg be observed and what is their magnitude?

## Methods

### Participants

For participant recruitment, a digital information letter was shared with local cycling and triathlon communities and further distributed by word of mouth. A sample of 12 male participants took part in the study (31.6 ± 10.9 years; body mass: 78.1 ± 12.9 kg; height: 179 ± 6 cm; body fat percentage: 14.4 ± 4.6%; adipose tissue thickness (ATT) left VL: 5.1 ± 2.1 mm; ATT right VL: 4.9 ± 2.2 mm; relative peak power output (PPO): 4.14 ± 0.6 W/kg; 10.0 ± 2.5 h of training per week; 7.1 ± 5.0 years of experience). A required sample size of 10 for the test–retest agreement of SmO_2_ was calculated using the G*Power software (version 3.9.1.7, Kiel, Germany) with a targeted power of *β* = 0.8, *α* = 0.05 and a correlation between repeated measures of 0.9 based on the test–retest correlations reported by (Crum et al. 2017). Race experience was required for inclusion. Furthermore, participants had to be healthy and non-smokers. Participants were excluded when taking medication affecting metabolic or cardiovascular performance. The dominant leg was determined using the ball kick test. Seven participants were cyclists, and five participants were triathletes. Based on PPO and training hours, the participants can be classified as *recreationally trained to well-trained* according to the classification proposed by De Pauw et al. (2013). Participants received information regarding the study design and the physical tasks beforehand. Written informed consent was attained before the first test. The study was conducted in accordance with the Declaration of Helsinki, and the protocol was approved by the local Ethics Committee (No. 23-19).

### Design and procedures

#### Experimental design

All participants performed three incremental step tests separated by 2–7 days. The tests were performed in the lab of the Institute of Sports and Preventive Medicine of Saarland University at the same time of the day on each occasion (± 1 h). The participants were instructed to refrain from fatiguing (long or vigorous) exercise 24 h before the tests and to shave their thighs thoroughly to rule out any impact of body hair on the measurements (Barstow 2019).

#### Pre-exercise protocol

At the beginning of the first visit height, body weight, body fat percentage, skinfold thickness at the VL on both legs as well as training and competition history in the sport were assessed. The Moxy devices together with the light shields provided by the manufacturer were placed on the VL of both legs approximately halfway between the greater trochanter and the lateral epicondyle of the femur (Crum et al. 2017). The location of the device was marked using a black permanent marker to ensure identical placement during the following trials.

#### Exercise protocol

The cycling step test was performed on the participants own bike mounted on an electronically braked cycle ergometer (Cyclus2, RBM elektronik-automation GmbH, Germany). The protocol started at 1.0 W/kg body weight and every 5 min the resistance was increased by 0.5 W/kg. The test was terminated when voluntary exhaustion was reached. The participants were asked to cycle at their preferred cadence and the supervising sport scientist visually controlled that the same cadence was maintained throughout and between the tests to avoid confounding effects of variable cadence on SmO_2_ (Skovereng et al. 2016). Participants had the option to use an electrical fan for air flow. The settings were replicated between trials to rule out any differences in cooling. The exercise protocol with five-minute stages was chosen to allow attainment of a SmO_2_ steady state. The starting intensity and increments in relation to the body weight were chosen to allow for better comparison between participants.

### Measures

#### Adipose tissue thickness

A skinfold caliper (British Indicators LTD, England) was used to access body fat percentage using the sum of 10 skinfolds method (Parizkova 1961) and skinfold thickness at the VL muscle. Adipose tissue thickness (ATT) was calculated as follows: Skinfold thickness × 0.5 (Barstow 2019).

#### Muscle oxygenation

Two portable, commercially available continuous-wave NIRS devices (Moxy Monitor, Fortiori Designs LCC, US) were placed on the VL of the dominant (DOM) and the non-dominant leg (NDOM) to measure SmO_2_. The standard settings for recording (0.5 Hz, smoothing enabled) were used. Data was recorded on a standard bike computer (Edge 530, Firmware Version 9.73, Garmin, US, Kansas) using two Connect IQ data fields (version 2.14) provided by the manufacturer. The Moxy uses one light emitting diode sequentially sending light waves in four different wavelengths (630–850 nm) into the underlying tissue. 2 detectors, spaced 12.5 mm and 25 mm from the emitter, measure the reflected light and a proprietary algorithm to overcome limitations of the modified Beer-Lambert equation is applied (Feldmann et al. 2019). As continuous wave NIRS relies on the assumption that the differential path length factor and the losses due to scattering are constant, only a quantitative measure of muscle oxygenation can be provided (Barstow 2019). The algorithm is intended to isolate oxygenation of muscle tissue from superficial tissue layers and therefore the term SmO_2_ instead of tissue oxygen saturation is used (Feldmann et al. 2019, 2022).

### Data analysis

The .fit files containing the NIRS data were imported into Golden Cheetah (version 3.6, https://www.goldencheetah.org). To compare the last minute of each stage, laps were created for the average SmO_2_ value for DOM and NDOM. All data were entered into SPSS (IBM SPSS Statistics Version 29.0.0.0, IBM, US, New York) for further analysis. All figures were created using R Statistical Software (v4.3.2, R Core Team, 2023) using the ggplot2 package (v3.4.4, Wickham, 2016).

#### Statistical analysis

First, to assess absolute reliability, a two-way repeated measures ANOVA was performed to estimate the standard error of measurement (SEM) as the square root from the mean square error term (Atkinson and Nevill 1998; Hopkins 2000; Weir 2005) for left and right VL. Sphericity was assumed when Mauchly's test returned an *α* > *0.05*. If sphericity was present, the Greenhouse–Geisser correction was used (Field 2017). For relative reliability, the two-way random intraclass correlation coefficient (ICC) for single scores (model 2,1, based on the nomenclature by Shrout and Fleiss Koo and Li 2016; Weir 2005)) was calculated for each workload and for DOM and NDOM, respectively. The minimal detectable change (MDC) for 95% confidence intervals was calculated using the formula:

$$MDC \, = \, SEM \times 1.96 \times \sqrt 2$$

(Weir 2005). A subgroup analysis for ICC and SEM was performed excluding participants with ATT > 7 mm as it has been shown that adipose tissue thickness above 7 mm has an influence on SmO_2_ values (McManus et al. 2018). A three-factor ANOVA (Trial*Side*Stage) was used to investigate the differences between DOM and NDOM for each power output. Additionally, the bias and 95% limits of agreement between the SmO_2_ values of DOM and NDOM were investigated with the modified Bland–Altman method for repeated measures with varying true values (Bland and Altman 2007). A paired-samples t-test was used to investigate the difference between skinfold thickness at the DOM and NDOM VL. The level of significance was set to *α* = *0.05* for all tests.

## Results

**Fig. 1:**
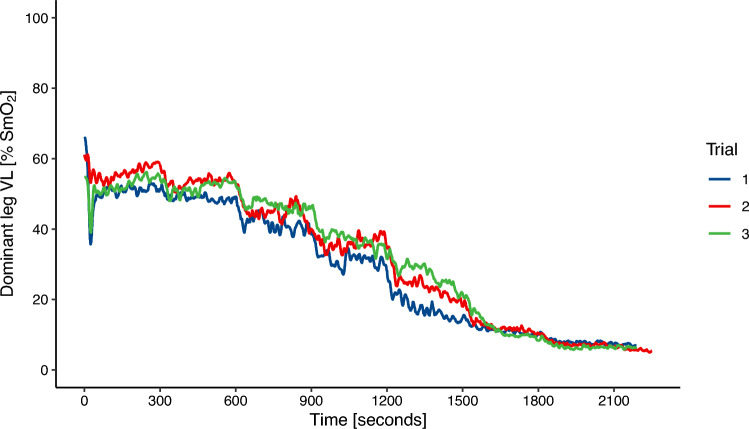
Experimental NIRS recordings of one participant. SmO_2_ time course data for the dominant leg is presented for all 3 trials

**Fig. 2:**
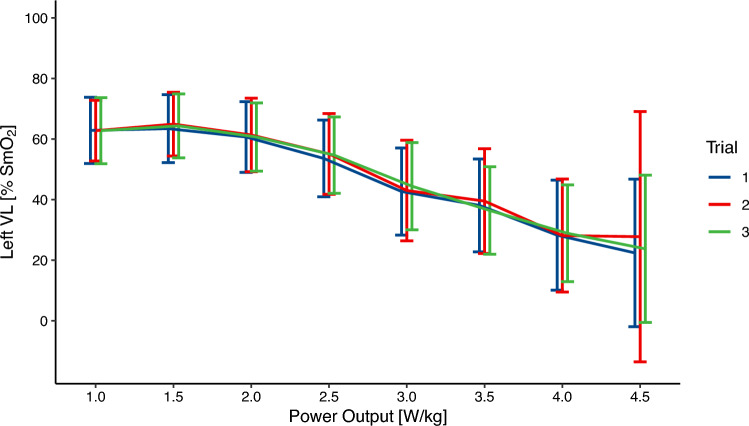
SmO_2_ values of the left vastus lateralis for each power output and trial. Presented as mean ± 95% confidence interval

**Fig. 3:**
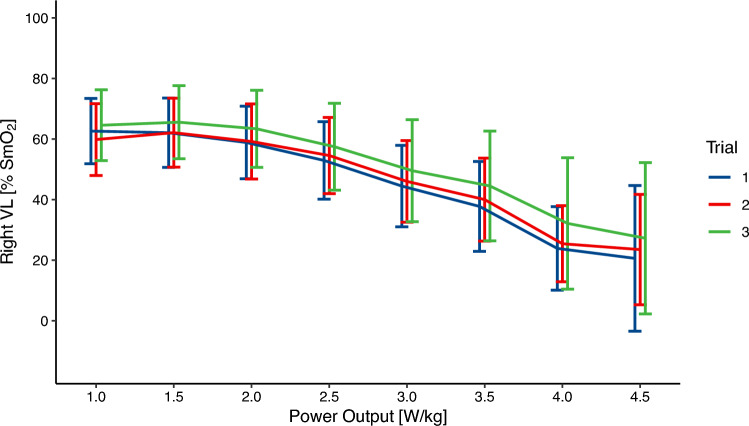
SmO_2_ values of the right vastus lateralis for each power output and trial. Presented as mean ± 95% confidence interval

Figure 1 presents exemplary SmO_2_ time course data for one participant and all trials. Two participants repeated one test due to 1) a freeze of the Garmin data fields and 2) large dropouts in data transmission. One data set for the left and two data sets for the right leg were excluded due to implausible muscle oxygenation kinetics like sudden large drops or increases in SmO_2_ that were only present in the SmO_2_ data of one leg. These tests could not be repeated due to time constraints of the participants. Furthermore, the data of seven individual stages from three participants had to be excluded due to dropouts. These dropouts occurred at workloads at or above 3.0 W/kg. The remaining data of these tests was still used. In total, 9 out of 76 individual data sets, or 12% of all cases, were either excluded or incomplete. 2 Participants had an ATT > 7 mm and were excluded for additional sub-analysis. The average SmO_2_ values for each trial are presented in Figs. 2 and 3 for the left and right VL, respectively.

### Absolute reliability

The SEM as well the MDC for each step and left and right VL are presented in Table 1. The SEM ranges from 5–9% SmO_2_ with an average SEM of 6% SmO_2_. The MDC ranges from 14 to 21% SmO_2_. Both SEM and MDC were similar for the subgroup analysis.

**Table 1:** Absolute and relative reliability for each power output. 95% Confidence Intervals are provided in square brackets when applicable. Values for sub-analysis are in brackets

Power Output	Side	n	ICC	SEM	MDC
(W/kg)				(% SmO_2_)	(% SmO_2_)
1.0	L	11 (9)	0.79 [0.53, 0.93] (0.86 [0.63, 0.96])	8 (6)	21 (17)
	R	10 (8)	0.87 [0.66, 0.99] (0.78 [0.45, 0.95])	7 (7)	18 (18)
1.5	L	11 (9)	0.84 [0.62, 0.95] (0.87 [0.66, 0.97])	7 (6)	19 (16)
	R	10 (8)	0.88 [0.71, 0.97] (0.79 [0.48, 0.95])	6 (7)	17 (18)
2.0	L	11 (9)	0.90 [0.75, 0.97] (0.90 [0.72, 0.97])	6 (6)	17 (16)
	R	10 (8)	0.94 [0.83, 0.98] (0.90 [0.70, 0.98])	5 (5)	14 (14)
2.5	L	11 (9)	0.92 [0.80, 0.98] (0.93 [0.80, 0.98])	6 (5)	16 (14)
	R	10 (8)	0.94 [0.84, 0.98] (0.91 [0.74, 0.98])	5 (5)	14 (14)
3.0	L	10 (9)	0.90 [0.74, 0.97] (0.90 [0.73, 0.97])	8 (7)	21 (20)
	R	10 (8)	0.94 [0.84, 0.98] (0.92 [0.76, 0.98])	6 (5)	16 (15)
3.5	L	9 (8)	0.89 [0.71, 0.97] (0.89 [0.68, 0.97])	8 (8)	21 (22)
	R	9 (7)	0.94 [0.82, 0.98] (0.91 [0.71, 0.98])	6 (6)	17 (16)
4.0	L	7 (7)	0.85 [0.57, 0.97] (0.85 [0.57, 0.97])	9 (9)	24 (24)
	R	6 (6)	0.89 [0.61, 0.98] (0.89 [0.61, 0.98])	6 (6)	18 (18)
4.5	L	4 (4)	0.92 [0.61, 0.99] (0.92 [0.61, 0.97])	6 (6)	16 (16)
	R	4 (4)	0.83 [0.32, 0.99] (0.83 [0.32, 0.99])	7 (7)	18 (18)
Average			0.89 [0.68, 0.97] (0.88 [0.64, 0.95])	6 (6)	18 (17)

*L* left vastus lateralis, *R* right vastus lateralis, *ICC* intraclass correlation coefficient, *SEM* standard error of measurement, *MDC* minimal detectable change, *SmO*_*2*_ muscle oxygenation

### Relative reliability

The ICCs for the different power outputs and left and right VL are reported in Table 1. The average ICC is 0.89 and individual ICCs range from 0.79 to 0.95 and were lower for lower workloads. The ICC in the sub-group analysis was similar, ranging from 0.78 to 0.95 and being 0.88 on average.

### Comparison of the dominant and non-dominant leg

**Fig. 4:**
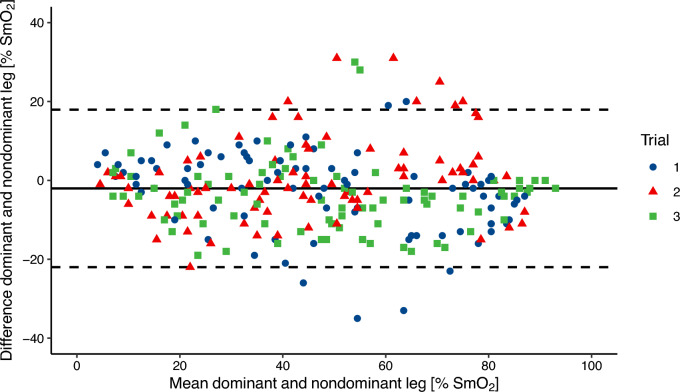
Bland-Altmann plot comparing the average SmO_2_ value of the last minute of the dominant and non-dominant leg VL for all participants and completed stages (−2.0% ± 19.9% (bias ± LoA))

The difference between the skinfold thickness of the DOM (*M* = *10.13, SE* = *1.33*) and NDOM (*M* = *9.78, SE* = *1.17*) VL was 0.36 mm, 95% CI [−0.68, 1.40] and not significant: *t(11)* = *0,754, p* = *0.47*. The three-factor ANOVA revealed no statistically significant main effects for side and trial as well as no significant interaction effects (*p* > 0.05). Figure 4 shows the Bland-Altmann plot comparing SmO_2_ values between DOM and NDOM. Mean bias was -2.0% and 95% confidence limits of agreement adjusted for repeated measures were −21.9% and + 17.9%.

## Discussion

The goal of this study was to investigate the reliability of SmO_2_ during cycling at different intensities and compare SmO_2_ between the DOM and NDOM leg. It was assumed that the average SmO_2_ in the last minute of each stage represents a steady-state behaviour and, thus, measurements can be interpreted as day-to-day variability of SmO_2_ during steady state exercise. For practitioners, coaches, and athletes the SEM provides a useful index for the reproducibility. In this study, the SEM ranges from 5 to 9% SmO_2_ dependent of the workload and was on average 6% SmO_2_.

This finding is highly similar to the reported SEM of Yogev et al. (2023a) of 5–7% SmO_2_ for standardized workloads for two similar, but intermittent cycling tests. The finding that the absolute reliability is similar during different intensities is in contrast to the results reported by Crum et al. (2017), who observed an increase in CV with increasing power output. However, The CV should be used when heteroscedasticity is present (Atkinson and Nevill 1998) and is less suitable for homoscedastic data as present here. After testing for heteroscedasticity, the SEM was chosen as more appropriate measure. Yogev et al. (2023a) also concluded that the SEM is more suited for homoscedastic SmO_2_ data.

The ICC reflects the ability to differentiate between individuals (Weir 2005) and using typical cut-offs, the ICCs obtained in this study, ranging from 0.78 to 0.95, indicate good to excellent relative reliability (Koo and Li 2016). The ICC values obtained in this study are very similar to the values reported by Crum et al. (2017) of 0.77–0.92 as well as Yogev et al. (2023a) of 0.81–0.90. Contreras-Briceño et al. (2019) state comparable ICCs for an incremental running test (0.95–0.97 for the VL and 0.84–0.93 for the intercostal muscles).

The MDC can be used to decide if an observed difference between two measurements can be considered real (Weir 2005). In this study the MDC ranges from 14 to 21% SmO_2_ and was on average 18% SmO_2_, implying that one can say with 95% certainty that the difference between two measurements under similar conditions is real if the SmO_2_ value for a specific power output differs by at least 18% SmO_2_. When considering directional changes, e.g. improved muscle oxygenation at the same power output, Hopkins (2000) illustrates that using 95% confidence intervals result in a 97.5% probability that the improvement is real. He points out that this amount of certainty is impractical in high-performance sports as it circumvents making any decisions for future training modifications. Applying this rationale, using a difference of half the MDC leaves an 84% probability that the improvement is real. In this case, depending on the workload, a meaningful difference would be in the range of 7–11%.

After excluding two participants with ATT values between 7.7 and 8.8 mm at the VL, both ICC and SEM remain mostly unchanged. Due to the small number of participants with ATT values only slightly above the maximum recommended value of 7 mm by McManus et al. (2018), no prediction can be made whether higher ATT has an impact on the reliability of muscle oxygenation at the VL during steady-state cycling. While ATT mostly explained between-subject differences at rest in their study, the impact of ATT on reliability was not investigated.

If NIRS can be used to delineate different power outputs, it could be used to prescribe and control exercise intensities. Between the power outputs of 1.0 to 2.0 W/kg the average SmO_2_ was almost constant (see Figs. 2, 3). At the same time, the SEM is about 7% SmO_2_ and therefore higher than the differences between 0.5 W/kg different power outputs. Between the power outputs of 2.0 W/kg to 4.0 W/kg the average SmO_2_ value drops by about 10% between stages while the SEM is approximately 6–7%. Between the last two stages the difference in SmO_2_ is smaller and around 4%. This is slightly smaller than the SEM of around 6% for these power outputs. This means that the SEM is higher than the difference in SmO_2_ between stages for low and high power outputs. In this sample, for power outputs in the range of 2.5 to 4.0 W/kg the SEM is smaller than the difference between mean SmO_2_ values, indicating that in this range SmO_2_ can be better used to differentiate between power outputs. However, due to different levels in fitness between participants, it is not possible to draw conclusions about the exercise intensity domain at these workloads. Similarly, Bonilla et al. (2022) did not find a difference in SmO_2_ between neighbouring steps of a graded exercise test, but SmO_2_ was significant different between maximal fat oxidation, the first as well as the second ventilatory threshold.

In addition to the investigation of the reproducibility of SmO_2_, left and right SmO_2_ were compared to explore side differences. No systematic difference between DOM and NDOM was detected. This is confirmed by the Bland-Altmann plot showing a small bias of 2% lower SmO_2_ for the dominant leg. However, the wide limits of agreement (~ ± 20% SmO_2_) show that left and right values can differ substantially. No significant side differences of SmO_2_ are in contrast with reported bilateral differences in power output and a reported roughly 25% higher deoxy[heme] amplitude in the dominant leg during ramp tests (Iannetta et al. 2019). Similar to our findings, Reinpõld and Rannama (2023) found that differences in bilateral desaturation onset kinetics were unrelated to leg dominance. The results indicate that leg dominance does not explain side differences in SmO_2_. The unexplained differences could be explained by measurement error. As the same device used was on the same leg in this study, further studies are needed to investigate if this is due to a between-device error or biological variability.

One unexpected finding of this investigation was that 12% of the individual data sets were incomplete or had to be excluded completely. Possible reasons for the observed dropouts are movement artifacts or tissue ischaemia as pointed out by Crum et al. (2017) or interference in the wireless data transmission. Using a bike computer placed in close proximity to the rider (~ 1 m) to record the data replicates how the devices typically would be used. Anecdotally, no dropouts during outdoor cycling were observed with the same bike computer. Based on the loss of data in ≥ 10% of cases it can be recommended to use a second Moxy on the opposite limb as a backup in case of faulty or missing data.

### Limitations

This study is, as any research, not without limitations. First, a small sample of only 12 participants was used (Atkinson and Nevill 1998). The findings need to be replicated with a larger number of subjects to further investigate differences between ATT and muscle oxygenation. Future studies should also include female participants as female athletes typically have higher adipose tissue thickness than men (McManus et al. 2018). Some research exists indicating that Moxy-derived SmO_2_ and its kinetics differ between sexes (Espinosa-Ramírez et al. 2021; Sendra-Pérez et al. 2023), but the effect of sex or higher ATT on the reliability of SmO_2_ remains unclear. Nevertheless, to the authors best knowledge this is the first study investigating the reliability of the Moxy device using 3 trials. The training and exercise regime between trials was not strictly controlled, which might have impacted the reliability negatively. In turn, this could also be considered a strength of the study design, as it might better reflect real-world conditions where not every training session is performed well rested and under identical conditions. One major limitation is that the results of this study can not be transferred to different sports or muscles. Finally, only SmO_2_ was investigated, as it has a higher practical relevance than total[heme], which also seems to be harder to interpret (Barstow 2019). Future studies should try to answer if different Moxy devices can be used interchangeably and if the SmO_2_ values of the opposite limbs are comparable.

## Conclusion

This study demonstrates that Moxy-derived muscle oxygenation values during an incremental cycling test are associated with good-to-excellent relative reliability determined using the ICC and an average SEM of 6% SmO_2_. These results are in line with previous research investigating the test–retest reliability of the Moxy device. Acceptability of the SEM as a measure of reproducibility can only be assessed with respect to the analytical goal. When the goal is to target a specific intensity, the Moxy was only able to delineate 0.5 W/kg differences between 2 and 4 W/kg in this sample. Thus, it cannot be recommended to use the absolute SmO_2_ value measured by Moxy for a precise intensity control. In order to detect changes in SmO_2_ between two measurements, a difference of at least 9% SmO_2_ needs to be observed to consider the improvement real with an 84% probability. Wide limits of agreement for side differences were detected in this sample, which could not be explained by leg dominance. Practitioners have to be cautious comparing SmO_2_ values between dominant and non-dominant leg VL. Therefore, it can be recommended to use the same device placed on the same leg and muscle to reduce the impact of between-device and side-specific differences.

## Data Availability

The datasets generated during, and the current study are available from the corresponding author on reasonable request.

## Abbreviations

ANOVA: Analysis of variance
ATT: Adipose tissue thickness
CV: Coefficient of variation
Deoxy[heme]: Deoxygenated hemoglobin and myoglobin
DOM: NIRS device placed on the dominant VL
ICC: Intraclass correlation coefficient
MDC: Minimal detectable change
NDOM: NIRS device placed on the non-dominant VL
NIRS: Near-infrared spectroscopy
Oxy[heme]: Oxygenated hemoglobin and myoglobin
PPO: Peak power output
SEM: Standard error of measurement
SmO_2_: Muscle oxygen saturation
Total[heme]: Total hemoglobin and myoglobin
VL: Vastus lateralis muscle
W/kg: Watt per kilogram bodyweight
